# Dominant transcript expression profiles of human protein-coding genes interrogated with GTEx dataset

**DOI:** 10.1038/s41598-022-10619-9

**Published:** 2022-04-28

**Authors:** Kuo-Feng Tung, Chao-Yu Pan, Wen-chang Lin

**Affiliations:** 1 Institute of Biomedical Sciences, Academia Sinica, Taipei, 115 Taiwan, R.O.C.; 2 Institute of Biomedical Informatics, National Yang Ming Chiao Tung University, Taipei, Taiwan, R.O.C.

**Keywords:** Genetic databases, Transcriptomics

## Abstract

The discovery and quantification of mRNA transcripts using short-read next-generation sequencing (NGS) data is a complicated task. There are far more alternative mRNA transcripts expressed by human genes than can be identified from NGS transcriptome data and various bioinformatic pipelines, while the numbers of annotated human protein-coding genes has gradually declined in recent years. It is essential to learn more about the thorough tissue expression profiles of alternative transcripts in order to obtain their molecular modulations and actual functional significance. In this report, we present a bioinformatic database for interrogating the representative tissue of human protein-coding transcripts. The database allows researchers to visually explore the top-ranked transcript expression profiles in particular tissue types. Most transcripts of protein-coding genes were found to have certain tissue expression patterns. This observation demonstrated that many alternative transcripts were particularly modulated in different cell types. This user-friendly tool visually represents transcript expression profiles in a tissue-specific manner. Identification of tissue specific protein-coding genes and transcripts is a substantial advance towards interpreting their biological functions and further functional genomics studies.

## Introduction

With the advancement of next-generation sequencing (NGS) platforms, unprecedented progress has been achieved in the fields of biology and medicine^1,2^. NGS platform is particularly essential in the advancement of modern genomic studies that mainly focus on determining genome sequences and deciphering the significant genome features of millions of nucleotide sequences. Genome sequence determination is now the simplest part of genome researches. However, obtaining the comprehensive annotations on all protein-coding gene loci and their gene structures are still challenging^3^. With the introduction of deep learning-based artificial intelligence (A.I.) machine learning approaches, exon structure prediction accuracy has improved^4,5^. Nevertheless, this is not enough to fully comprehend the molecular mechanisms in terms of protein-coding gene modulations. In humans and other higher organisms, manual interpretation and experimental evidence are yet needed to completely decipher transcribed mRNA transcripts of protein-coding genes^6^; this often involves the generation of alternative transcripts by using complex mRNA transcription maturation mechanisms^7^. Currently, precisely authenticating all possible alternative transcripts of protein-coding genes is still difficult because of the limitations of NGS platforms. Furthermore, diverse tissue expression modulations of these alternative transcripts complicate the authentication process. Protein-coding genes are regulated by developmental temporal programs and restricted tissue spatial patterns in addition to their common cellular physiological functions^8,9^. Because this is a complicated process with variations occurred in different genes, visual informatic tools are desirable to carefully investigate alternative transcript expression profiles in different tissues.

Although the sequencing portion of human genome project has been accomplished^10^, the exact human protein-coding gene structures and their mRNA transcript expression modulations would need to be thoroughly investigated in different tissues^11^. Therefore, we aim to develop a user-friendly web tool for exploring the tissue expression profiles of the alternative transcripts in human protein-coding genes. We used the NGS dataset from the Genotype-Tissue Expression (GTEx) project^12^; it is a well-known international consortium that provides essential research resources on genetic variations and global RNA expression data in multiple normal tissues. The GTEx project aims to create datasets for the systematic evaluation of genetic variations and examine their relationship with gene expression in multiple tissues^13,14^. In the current GTEx data release (V8 version), 54 human tissue subtypes are available. We believe this is a helpful gene expression dataset containing information on various tissue types and a better unified data resource than earlier datasets for the evaluation of tissue expression variations in alternatively transcripts. The use of a single gene expression dataset can help prevent complicated mRNA isoform quantification problems in NGS transcriptome analysis pipelines that occur if heterogenous sources are used. Besides, few databases provide transcript-level alterations with limited spliced transcript information and visualization for users^15^.

Therefore, GTEx provides an exceptional resource through the study of transcriptome among various normal tissues. Tissue-specific transcript expression profiles could vary among different tissues for certain genes during development and oncogenesis. Previous tissue expression profile studies often used expression information at the gene level^16^, thus, it is desirable to have graphical visualization tools to interactively examine the top-ranked transcript expression in diverse tissue types. We hypothesized that such alternative transcript modulations would have biological significance in protein product expressions, and biomedical researchers would be benefited from visual bioinformatic tools on these data.

Previously, we generated a web tool (TREGT) for visually illustrating the expression information on top-ranked transcripts of protein-coding genes using the GTEx dataset^17^. One can easily inspect modulations about expressed transcripts of one human protein-coding gene by their ranks as well as ratios among different tissue types. It is useful to observe switch events of top-ranked transcripts in certain protein-coding genes, which would implicate particular modulations on selected transcripts. While this web tool also provides visual tissue expression profiles on human protein-coding genes, it is lacking tissue expression level comparison information to specifically recognize tissue-specific genes or transcripts. There are needs to interrogate the tissue expression profiles on particular mRNA transcripts, since tissue-specific gene or transcript modulation would implicate distinctive biological functions in selected tissue types. Thus, we would like to provide an improved tool for interrogating the representative tissue expression profiles of human protein-coding genes. This new database would enable researchers to explore the top-ranked transcript expression profiles in different tissues as well as identification of significantly expressed genes or transcripts in selected tissues.

## Results

### Protein-coding transcripts in normal tissues

Studies have used the GTEx dataset for the analysis of alternative transcripts to systematically determine their expression profiles in human noncancer tissues^8,14^. The GTEx dataset was mainly used to avoid dysregulated expression information regarding cancer cells, and GTEx can provide outstanding tissue expression information from many different human tissue subtypes^13^. Version 8 of the GTEx dataset was mapped to the 199,324 transcripts and 58,219 genes in the GENCODE 26 human reference set. Within this dataset, there are 150,749 mRNA transcripts belonging to 19,591 protein-coding genes and we mainly interrogate protein-coding genes in this study. Among the 150,749 transcripts of protein-coding genes, there are various transcript types, including protein-coding, processed_transcripts, nonsense_mediated_decay, retained_intron etc. Only 80,354 transcripts are defined as actual protein-coding transcripts by the GENCODE transcript type feature. Herein, we started with 145,571 transcripts of protein-coding genes after removing further 5178 transcript records without expression values as described in the Methods section. A previous study indicated that approximately seven alternative spliced transcripts exist for human protein-coding genes^17^. Among all the alternative transcripts within a given protein-coding gene, only few transcripts are dominantly expressed. In most cases, the top five transcripts could occupy more than 90% of protein-coding gene expression levels. Subsequently, examination of the expression profiles of alternative transcripts in each protein-coding gene would reveal restricted expression patterns in dominantly expressed transcripts in selected tissue subtypes. Because transcripts could have different coding potential or regulatory significance (such as microRNA target sites)^18,19^, the tissue expression profiles of each highly expressed transcripts must be carefully examined. Therefore, we would like to develop a new bioinformatic tool to specifically examine the distinct expression profiles of dominant transcripts in protein-coding genes. We believe these dominant tissue specific transcripts, if translated into protein isoforms, could represent significant biological functions in particular tissues. We designated these tissue specific transcripts as representative tissue transcripts in this database.

### Representative tissue transcripts of protein-coding genes

To evaluate specifically expressed transcripts as representative tissue transcripts in various human tissues, we applied the standard score (Z-score) criteria to examine the expression data of 145,571 transcripts. The standard score is frequently used for outlier identification and calculated as the original raw data value minus the mean value divided by the standard deviation. Thus, the standard score illustrates the difference in the TPM value between a particular tissue expression amount and the mean average of all tissue types for any given transcript isoform. We applied the Z-score value of ≥ 3 as the cutoff to obtain specifically expressed transcripts in diverse normal tissues. The Z-score value of ≥ 3 indicated that the difference in expression is more than 3 standard deviations in that observed tissue type.

We then calculated the respective Z-score value for each transcript of protein-coding genes among different tissue types. Interestingly, we found that over 80% of the 145,571 transcripts had a Z-score value of ≥ 3 (117,114 records) in at least one of the 54 tissue types. Only 28,457 transcripts were commonly expressed transcripts in all tissue types, with their Z-score being < 3. This finding indicated that most of the human genes had distinctive tissue expression profiles. Among them, dominantly expressed transcripts found in a single tissue type were the major category, accounting for approximately 50% of tissue-dominant transcripts. In total, 77,606 transcripts had a Z-score of ≥ 3 in a single tissue class (Table 1). Furthermore, 35,865 transcripts were mutually represented in two tissue types. In addition, 3615 transcripts were present in three tissues and 28 respective transcripts were present in the maximum four tissues. Thus, most of the transcripts in protein-coding genes exhibited significant expression profiles in a selected tissue type or few tissue types. Furthermore, in the 54 GTEx tissue subtypes, some tissues were divided into diverse subregions or collected from distinctive body locations. Reasonably, some genes were found to be represented in multiple tissue subtypes, for example, adipose tissues (subcutaneous and visceral), brain tissues (cerebellar hemisphere and cerebellum), and skin tissues (suprapubic and lower leg).

**Table 1:** Numbers of tissue representing transcripts interrogated with their tissue expression Z-scores.

Tissues*	Numbers of transcripts	Numbers of transcripts (TPM ≥ 1)	Numbers of transcripts (TPM ≥ 10)	Numbers of transcripts (TPM ≥ 100)
Zero	28,457	14,558	3273	231
One	77,606	22,576	4447	423
Two	35,865	10,354	1663	112
Three	3615	912	145	9
Four	28	12	4	0
	145,571	48,412 (33.2%)	9532 (6.5%)	775 (0.5%)

Expressed transcript of protein-coding genes were calculated for their Z-core value in each tissue as described in the “Methods”.

*Numbers of tissues with Z-score >=3 for each transcript were noted (zero to four tissues)

### Tissue representative transcripts among top-ranked transcripts

**Figure 1:**
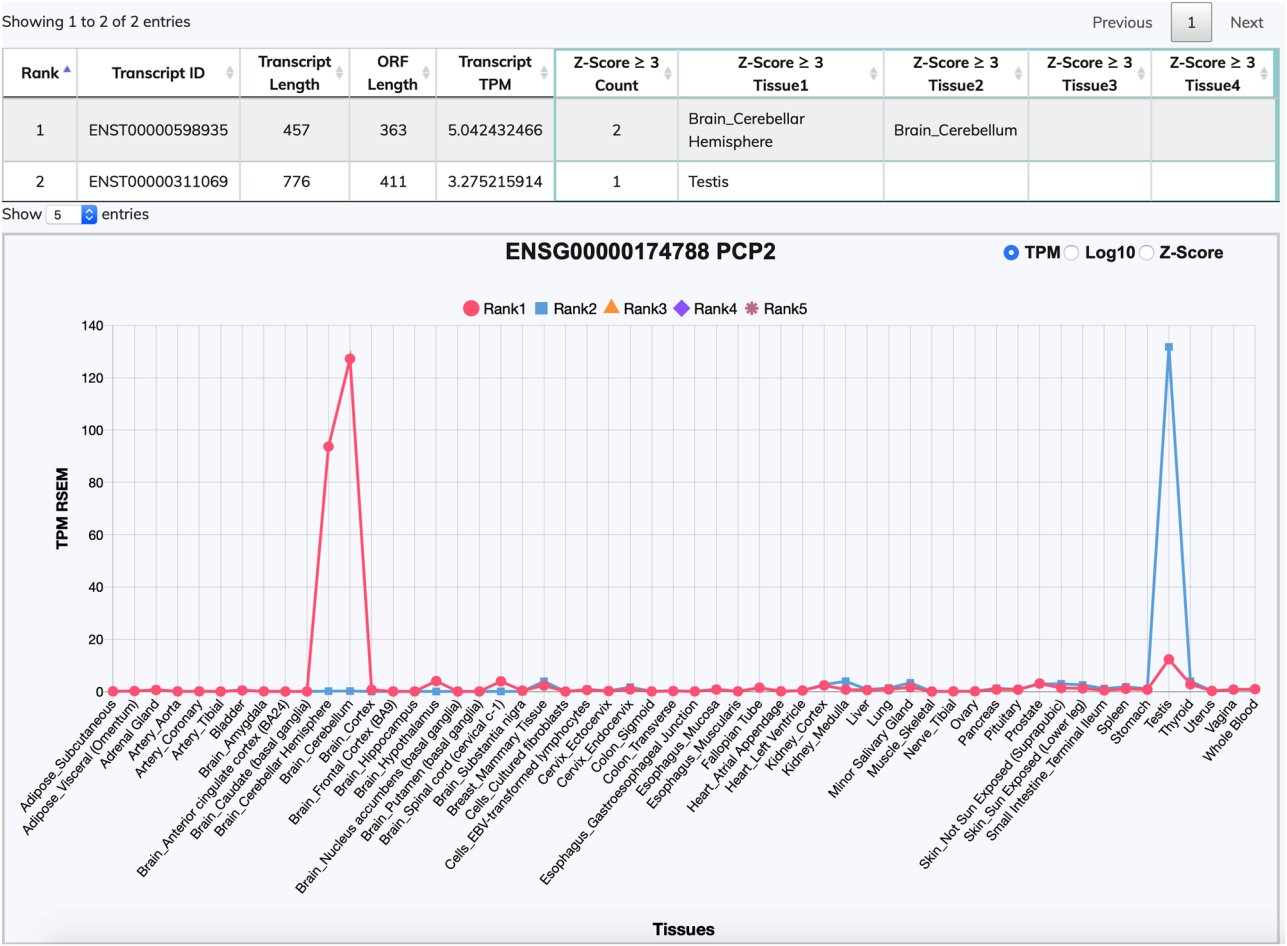
Tissue expression distribution of the *PCP2* gene. The human *PCP2* gene is a protein-coding gene for Purkinje cell protein-2, which has two transcripts. Rank1 transcript (ENST00000598935) is the major transcript expressed in brain cerebellum regions (cerebellar hemisphere and cerebellum), where all Purkinje neurons are located. Rank2 transcript (ENST00000311069) is the minor transcript isoform, which is highly expressed in the testis tissue. In the RTTPG user interface, the upper table provides additional information regarding gene and transcript IDs, gene name, transcript length, ORF length, TPM value, transcript Rank, and the represented tissue type for each transcript. In the tissue expression illustration panel below, users can see the tissue expression profile and change the expression scale from raw TPM values, log10 TPM values, and Z-score values.

For most protein-coding genes with alternative spliced transcripts, the majority of expression abundance are from the top five ranked transcripts^17^. Therefore, we further examined top-ranked transcripts in terms of their tissue expression profiles. In Supplementary Table 1, the coverage percentage of tissue-representative transcripts (Z-score of ≥ 3) was around 80% throughout Rank1 to Rank10 transcript classes. For instance, Rank1 transcripts has 15,197 tissue representative transcripts out of total 19,591 Rank1 transcripts. In general, different expressed ranked transcripts within the same gene (e.g., Rank1, Rank2, and Rank3 transcripts) had the same or closely related tissue expression profiles. These findings indicated that the tissue-specific transcript expression modulation is generally regulated at the gene locus level; therefore, similar tissue expression profiles were largely observed in most of the transcripts in the same gene loci. However, we did observe distinct tissue representation profiles among diverse transcripts in certain protein-coding genes. A demonstrative example is the Purkinje cell protein-2 (PCP2) gene, which has two alternative transcripts (Fig. 1). One transcript is ENST00000598935 with a strong expression profile in the brain (cerebellum and cerebellar hemisphere), whereas the other alternatively expressed transcript is ENST00000311069 and is mostly expressed in the testis tissue. This is an example of a typical altered tissue-specific expression between alternative transcripts in a gene.

In Supplementary Table 2, the average TPM expression value of Rank1 transcripts was 23.23 in one tissue category, whereas the TPM value of Rank2 transcripts was 6.14. On the contrary, the average expression value of universally expressed transcripts (Z-score < 3, zero tissue category) was 91.05 for Rank1 transcripts (Supplementary Table 2), indicating their intrinsic housekeeping gene nature with higher expression levels in a broad spectrum of tissue types. Among these genes in zero tissue category (Z-score < 3), MT-related genes (mitochondria genes) and the RPL gene family (protein-translation genes) exhibited the highest expression. DAVID functional analysis of these commonly expressed genes (genes with a Z-score of < 3 and an average TPM value of > 100) suggested their functional enrichment in translation initiation and ribosome functions (Supplementary Fig. 1).

**Figure 2:**
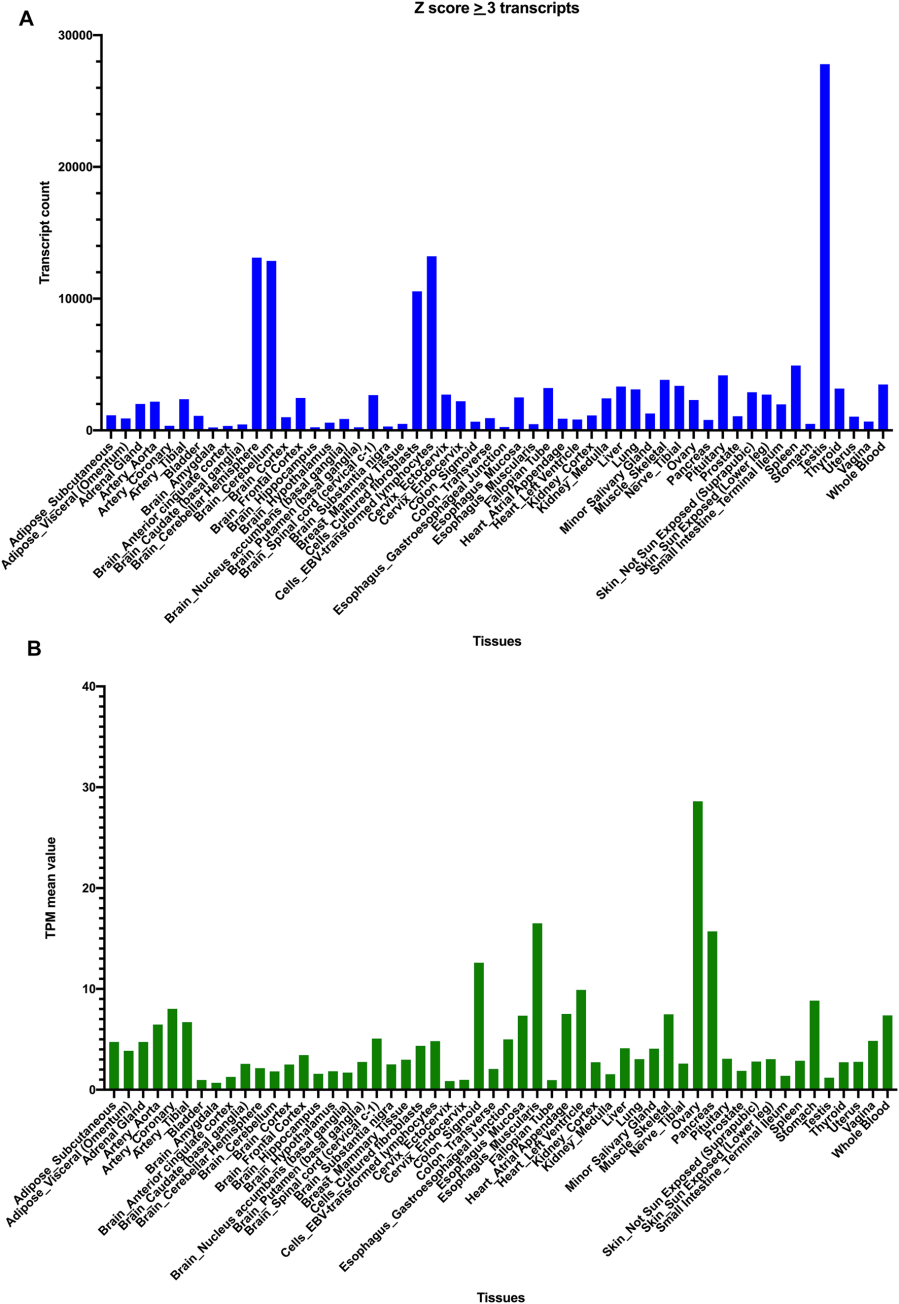
Tissue expression distribution of distinctive tissue expression transcripts. (**A**) The number of distinctive tissue expression transcripts (Z-score ≥ 3) in human tissues. (**B**) The average TPM expression values of distinctive tissue expression transcripts in human tissues.

Among the analyzed tissue expression results, the testis, cerebellum, and cerebellar hemisphere had the greatest numbers of dominant tissue expression transcripts (Fig. 2A). The testis had exclusive gene expression profiles for many protein-coding genes^20^. The cerebellum and cerebellar hemisphere had the most dominant tissue transcripts (approximately 13,000 transcripts). There are reported literatures on the enriched expression protein genes in both brain and testis tissues by large scale proteomic and transcriptomic studies^21,22^. Alternative splicing and polyadenylation events were also highly elevated in the brain and testis tissues^23,24^. Compared with some other brain regions, the amygdala, hippocampus, and putamen had less than 300 dominantly representative tissue transcripts. Thus, highly differential gene expression profiles were observed even in various tissue subregions (such as the brain), which implied the spatial gene expression patterns.

For individual transcript expression patterns (TPM levels), the overall profiles differed between tissue types. Hemoglobin subunit beta is the highest expressed tissue-dominant transcript (Rank1 transcript TPM = 5012.79). Although the testis had the maximum number of dominant tissue transcripts, their average expression level was only 1.2. The ovary had the highest TPM expression level of tissue transcripts, with an average TPM value of 28.6 (Fig. 2B). The pancreas had the second highest expression level of tissue transcripts (TPM = 15.7), with PRSS2 (trypsin-2 gene) being the most abundantly expressed gene. In some tissues, Rank1 transcripts were dominantly expressed transcript type, such as in the hippocampus.

**Figure 3:**
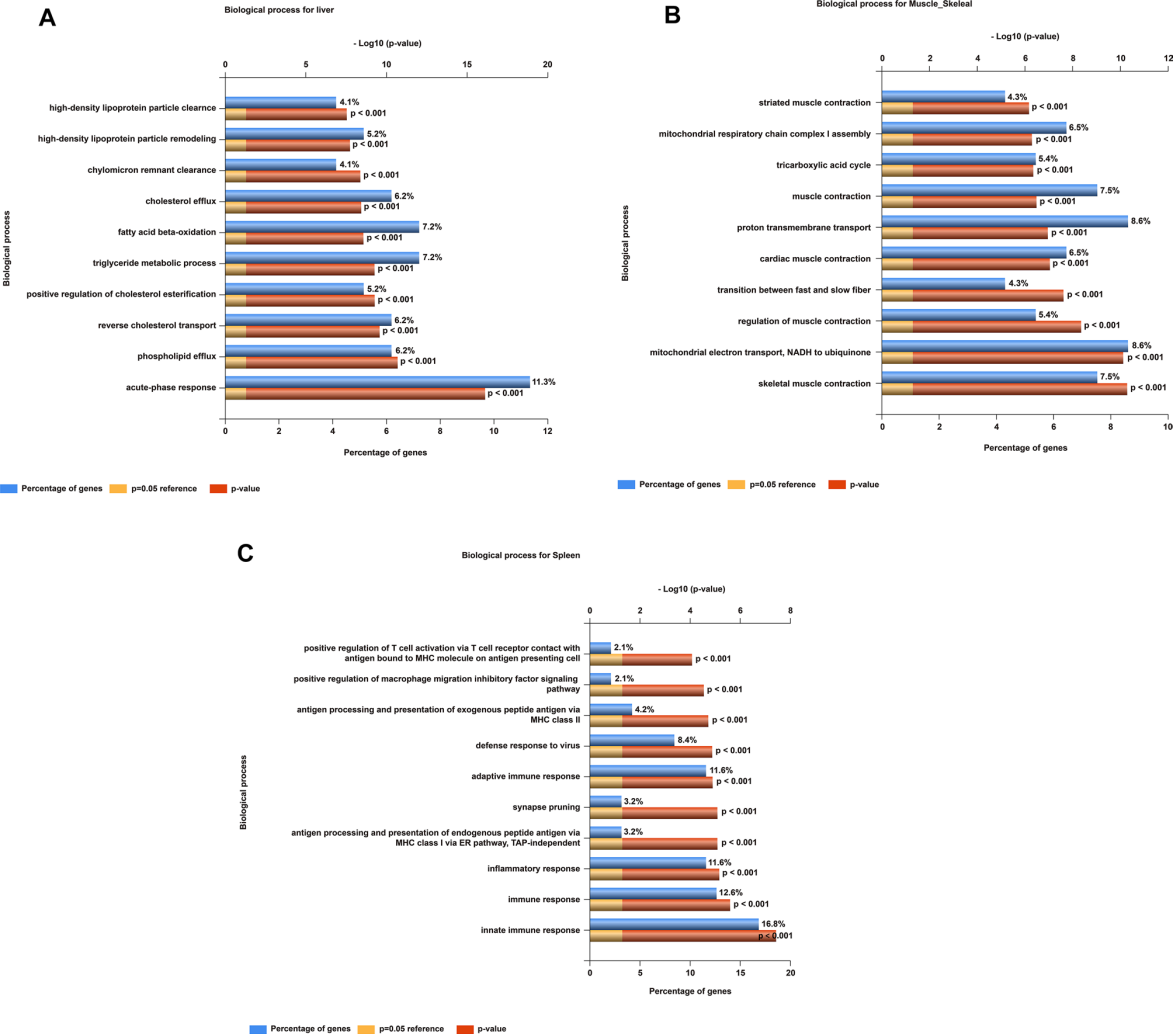
Pathway enrichment analysis for tissue representative protein-coding genes. Top 100 expressed genes from the following tissues were chosen for the FunRich enrichment analysis as described in “Methods”: (**A**) liver, (**B**) skeletal muscle, and (**C**) spleen. We used the GO-term biological process function for comparison in this study.

On the basis of the functional pathway enrichment analysis, we selected top 100 Rank1 transcripts from different tissue types to examine their functional significance. Among those abundantly expressed liver genes, the top 3 gene ontology (GO) terms were enriched in the acute-phase response, phospholipid efflux, and reverse cholesterol transport (Fig. 3A). Muscle genes were enriched in skeletal muscle contraction, mitochondrial electron transport (NADH to ubiquinone), and muscle contraction regulation (Fig. 3B). For the spleen tissue, the top 3 enriched functions were those involved in innate immune responses, immune responses, and inflammatory responses (Fig. 3C). Thus, these dominantly expressed tissue representative genes appeared to be highly correlated with known biological functions in selected tissues.

### User-friendly interface for examining tissue-dominant transcripts

We developed a user-friendly web tool to examine dominant tissue-representative transcripts of protein-coding genes in normal human tissues. Following the concepts of our previous database design, this representative tissue transcripts of the protein-coding gene (RTTPG) database specifically concentrated on the visualization of the tissue expression profiles of protein-coding genes. The GTEx dataset provides a unique gene expression resource that would be suitable for the functional investigation of alternative transcripts expression in noncancer tissues.

We mainly identified dominantly expressed transcripts by their Z-score values according to various tissue types by using an easy-to-recognize graphical illustration. We observed that the majority of significantly expressed transcripts were among the top-ranked transcripts. In the main web entry window, information regarding the top five ranked transcripts was provided to reduce the crowding of display information for genes with many transcripts (Fig. 1). We believe that this design would provide superior tissue expression profiles to biologists. However, an optional “rank selection” function can be used to examine all transcripts and top 10, top 5, top 3, besides the highest ranked Rank1 transcripts to fit research needs.

**Figure 4:**
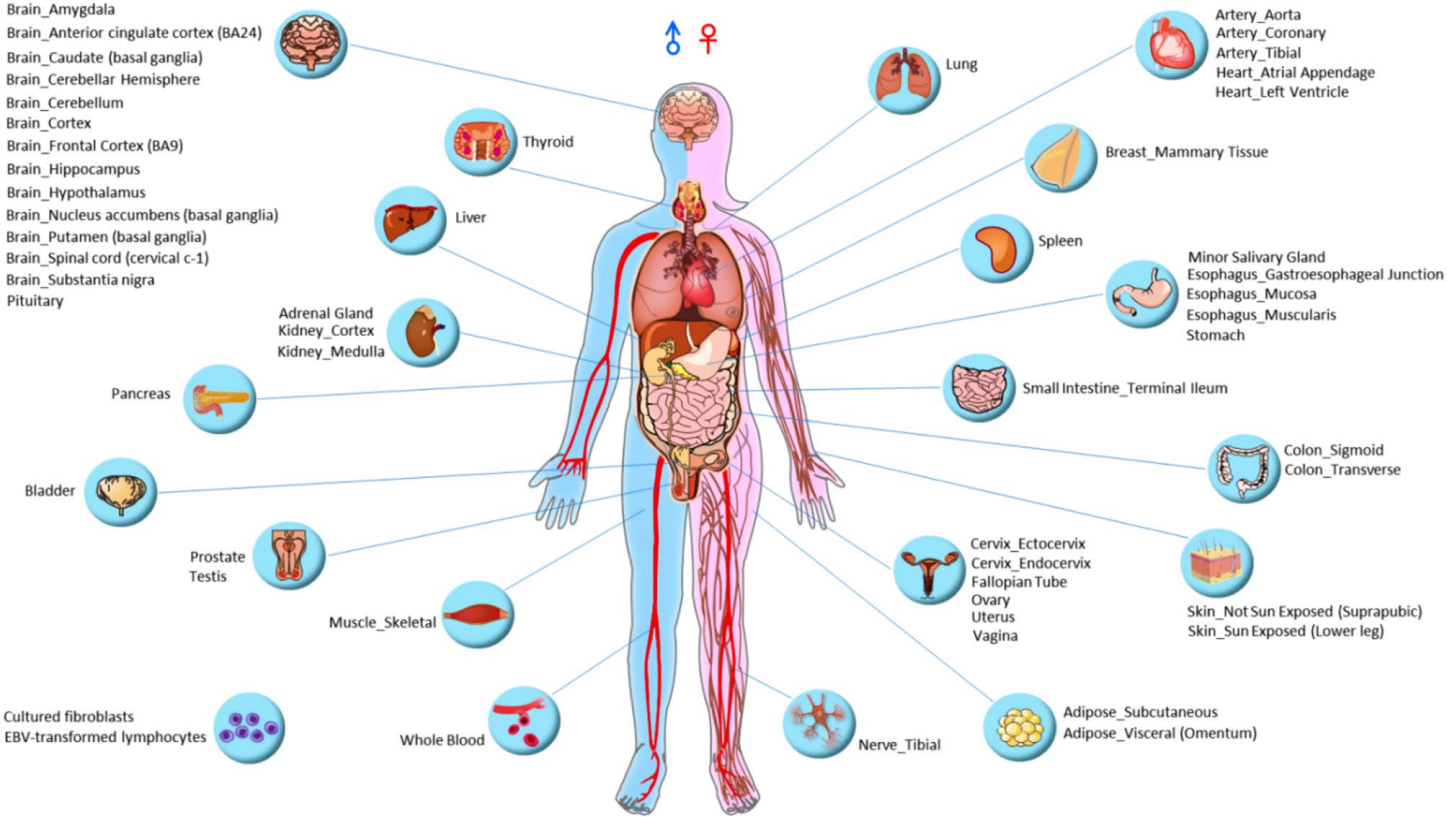
Web interface of the RTTPG database. We generated a graphic display interface for selecting representatively expressed transcripts in human tissues. In the default setting, Rank1 transcripts with a Z-score of ≥ 3 were designated for interrogations in the initial home page. Hovering the mouse over the tissue label text will show the number of representative transcript numbers. Users can click on any particular tissue icon label of their study interest. A new web table page will list the transcripts with a Z-score of ≥ 3 in that particular tissue. Users can further select any gene of interest for further interrogation for the alternative transcript expression in different tissue types shown in Supplementary Fig. 2.

At the beginning of the web page, users can first choose a particular tissue icon of their study interest (Fig. 4). A new web page will list the transcripts with Z-scores of ≥ 3 in that particular tissue. The features of the web page include gene ID, gene name, transcript count, transcript ID, rank of that particular transcript, transcript TPM value, coefficient of variation (CV), gene TPM, and Rank TPM% (Supplementary Fig. 2A). Sorting function is provided to all feature columns, and users can easily select features for further investigation. For interrogating any particular gene, users simply click on the gene ID link, and another new window will be displayed with details of the transcript or gene along with a graphic illustration of the top 5 ranked transcript expressions among various tissues.

In this graphic protein-coding gene expression information page, besides essential gene information, summarized gene functions are listed including protein class; molecular function and disease involvement. Users can further compare the features of all alternative transcripts based on their expression rank, transcript length, ORF length, and dominantly expressed tissues (Z-score ≥ 3) in the transcript list section. In the expression graphic display, the GTEx tissue expression levels of the top 5 ranked transcripts are displayed (Supplementary Fig. 2B). Users can analyze expression information by using TPM or Z-score values, and Log2, Log10 buttons are available for changing the TPM expression scale to log scale to observe lowly expressed transcripts. If the user holds the mouse over any given data point, a pop-up text will display the expression TPM value or Z-score value of particular ranked transcript. On the top right corner, the TREGT link displays additional transcript rank information from our previous TREGT web database, which delivers additional transcript expression information and tools.

With this helpful web tool, users can freely examine any protein-coding gene of their interest for expression profiles in different tissue types and easily identify tissue-specific transcripts or genes. This observation is assisted by using the CV value sorting functions when determining tissue-representative transcripts following selecting the tissue icons. For example, AVP, HCRT, and PMCH genes are uniquely expressed in the hypothalamus tissue; CFHR2, MASP2, and AHSG genes are expressed in the liver tissue; BMP10, NPPA, and MYL4 genes are expressed in the heart tissue (atrial appendage); PGA5, PGA4, and GIF genes are expressed in the stomach tissue; and DEFA6, DEFA5, and FABP6 genes are expressed in the small intestine tissue. In addition, genes expressed in particular physiological systems, such as GUCA2B gene, which is expressed more in the small intestine, colon, stomach, and liver, can be observed with our web interface. Therefore, this RTTPG web tool could be beneficial for biomedical researchers in functional analysis.

## Discussion

With the advancement of the NGS technique, scientists have generated a large amount of transcriptome data. Increased transcriptome data have helped scientists to learn more about the expression patterns of human protein-coding genes^25,26^. Human protein-coding genes have more alternative transcripts than previously estimated^3^. However, due to the read length limitations of NGS platforms, studying all alternative transcripts, particularly expression profiles among various cell types, may be challenging. With accumulating transcriptome data, concerns regarding the expression and biological significance of these transcripts are arising. It is likely that transcriptional variations would occur inside cells, and not all transcripts would conduct proper biological functions. Intriguingly, recent proteomic studies on the expression and distribution of human protein isoforms have implied that only limited numbers of protein isoforms for protein-coding genes have been detected^22,27^. Using several proteomic datasets, Ezkurdia et al. suggested that the majority of protein-coding genes express only a main protein isoform^28^. The APPRIS database is an excellent web resource for identifying main principal protein isoforms of protein-coding genes in many species^29^. In a recent comprehensive study using proteomic and transcriptomic datasets, Rodriguez et al. demonstrated that tissue specific alternative splicing events (ASE) in the alternative splicing transcripts and subsequent protein isoform productions in human protein-coding genes^30^. This phenomena is highly conserved evolutionally in some genes and would have important functional implications. We also observed that overlapping significant tissue types of reported ASE genes with our RTTPG data. Furthermore, our web tool could be valuable in assisting visual interrogation of GTEx tissue expression profiles among different top-ranked transcripts.

This generates discrepancy and debate on the numbers of protein-coding transcripts and their translated products, especially concerns about the actual biological significance of alternative transcripts. ^31^ Although more alternatively expressed mRNA transcripts could be discovered using NGS sequence data, limited protein molecules were observed by using the proteomic platform data. This observation might be attributable to the sensitivity and throughput of the current shotgun-based mass spectrometry platform in the detection of all minor protein isoforms in tissues as well as the complicated issues in identification of posttranslational modified peptides. Some translated protein variants might have low abundancy or short half-life. Furthermore, another reason may be the distinctive expression profiles of different mRNA transcripts observed in only particular cell types^15^. It is likely that transcripts are preferentially expressed in certain cell or tissue types, this is particularly evident in developmental stages^9^, or pathological malignant conditions^32^. This point suggests the importance of investigating detail transcript expression in different tissue or cell types. Understanding the difference in expression between each transcript can provide more insights into putative functional effect on different transcripts in respective tissue types. The RTTPG web tool presented here would be beneficial for biologists to perform a thorough analysis of transcripts in many tissue types.

Besides expression levels, our web interface provides additional easy to read transcript information, especially the length and coding protein information. This feature is absent in most web databases. In a recent report, distorted transcription start and termination sites were the main classes in novel transcripts instead of conventional alternatively spliced exon–intron selection usages^33^. Thus, protein coding sequences might not be altered in most transcripts with alternative UTR regions. Furthermore, this opinion supports the findings of proteomic studies regarding a dominant protein isoform^28^. Our previous findings demonstrated that the dominant transcripts of protein-coding genes were the top-ranked transcripts and often represented the major expression transcripts for most protein-coding genes^17,34^. It is suggested that the transcriptome complexity of protein-coding genes may not be as high as estimated earlier based on the number of alternative transcript s identified by NGS reads. Moreover, not all top-ranked transcripts are protein-coding transcripts^17,34^. Many alternative transcripts are actually noncoding transcripts, and we observed that only 80,354 of 145,571 these transcripts annotated as protein-coding transcripts by the GENCODE biotype. Therefore, expressed transcript isoforms must be examined in more detail in addition to their tissue expression profiles. This further strength the applications of the RTTPG database.

## Conclusion

We have utilized the GTEx dataset to establish a web database for visualization of alternative transcripts expression in various normal human tissue subtypes. This web tool would be useful in analyzing distinctively expressed transcripts in a tissue specific fashion. Knowledge learned about the tissue specific expression profiles would be valuable in cellular function analysis for further single cell sequencing studies.

## Methods

### GTEx data processing

The Genotype-Tissue Expression (GTEx) Project was supported by the Common Fund of the Office of the Director of the National Institutes of Health, and by NCI, NHGRI, NHLBI, NIDA, NIMH, and NINDS^12^. The data used for the analyses described in this manuscript were obtained from the GTEx Portal on 05/20/20. The transcript expression data file (GTEx_Analysis_2017-06-05_v8_RSEMv1.3.0_transcript_tpm) was retrieved for our study. This public release dataset here do not contain participant information and also follow the NIH Genomic Data Sharing guideline^13^. Only the gene expression data and average values were used. We have then preprocessed the GTEx public dataset as described previously^17^. In brief, GTEx datasets were downloaded from their data portal website, which contained 54 different human tissue types from 948 donors. Some tissues were divided into subregions; notably, the brain tissue had the most subtypes^13,14^. GENCODE 26 (GRCh38 genome build) was the human gene and transcript annotation standard applied for the GTEx expression analysis pipeline—Version 8. The data file was first pre-processed using Python scripts to generate tissue based expression information according to each transcript and gene. Originally, total numbers of transcript records in this file was 199,324 records. We then removed 5178 transcripts without any expression information among all tissue samples. We used the GENCODE biotype labels to further classify the transcript and genes^35^. Non-coding genes and their downstream transcript records were filtered and removed. At this stage, we selected only 145,571 expressed transcripts from protein-coding genes for this study, and there are 19,591 protein-coding genes in this processed dataset.

### Ranking of transcripts and Z-score analysis

Due to the large numbers of data, the transcript expression information was initially processed and divided according to the different tissue subtypes using Python scripts. We then summarized the average expression values of each transcript within all protein-coding genes by respective tissue subtypes due to the unequal numbers of donor samples. The overall average expression information of every transcript was then tabulated from all GTEx tissues subtypes. Ranking of transcripts in protein-coding genes was mainly determined by their expression values. Accordingly, assignment of Rank1 transcript is annotated to the most abundantly expressed transcript. In some cases, the ORF length or transcript length information of particular transcripts were utilized if the average transcript expression TPM values were identical among transcripts. The transcript with longer ORF length or transcript length was preferably selected and annotated with higher order in Ranks. Expression characteristics of all ranked transcripts in each protein-coding genes were further collected and investigated as reported previously^17^. Python and R packages were used for subsequent statistical analyses^36^. For selecting distinctively expressed tissue transcripts, we used the standard-score (Z-score) values, which was computed as the raw data value in each tissue minus the average TPM value, divided by the standard deviation. We used the Z-score cutoff of ≥ 3 in order to select distinctively representative tissue transcripts. The Z-score value of ≥ 3 indicated that the difference in expression was larger than 3 standard deviations with a p value of 0.00135.

The Z-score value allow us to learn about the significant difference of each transcript in a given tissue type. For further identifying uniquely tissue representative genes or transcripts across all tissues, the coefficient of variation (C.V.) value sorting function was applied to easily reveal tissue-specific transcripts or genes in the gene list user interface. Higher coefficient of variation value would indicate a more unique tissue expression profile among all tissues.

The variance between the transcripts among different tissues would indicate the tissue-specific expression patterns.

### Functional assignment and enrichment analysis

The DAVID (Database for Annotation, Visualization, and Integrated Discovery) functional analysis was performed to identify functional enrichment classes^37^. The DAVID Bioinformatic Resources 6.8 (https://david.ncifcrf.gov) was used to obtain significantly enriched GO terms and Kyoto Encyclopedia of Genes and Genomes (KEGG) pathways. Statistical significance of such pathway enrichment analysis was set with p value of < 0.05. Selected genes were uploaded to the DAVID analysis pipeline, and default parameters were used for identifying enrichment clusters.

We used another published bioinformatic tool, FunRich (3.1.4), which is useful for functional enrichment and gene network analyses^38^. The software program was obtained from the FunRich web site (http://funrich.org). The top 100 genes of Rank1 transcripts from respective tissues were chosen for enrichment analysis. We used the GO-term biological process function for comparison according to published instructions.

### RTTPG web database construction

The RTTPG database was implemented using PHP language on an Apache webserver framework in conjunction with the MySQL database as described previously^39^. All transcript expression data on the webserver are stored in a flat file format and loaded into MySQL database for RTTPG web interfaces. In order to further investigate tissue representative transcripts, we provided the list of putative genes based on the tissue subtypes. Several important features of transcripts were displayed for users to examine their significance, including transcript TPM value and ORF region length. The graphic expression page then displayed the 54 GTEx tissue expression information on top five Ranked transcripts. Users can analyze expression information by using TPM or Z-score values. Furthermore, additional gene functional annotations on protein-coding genes were retrieved from the Human Protein Atlas database and processed^22^. We matched the GTEx and Human Protein Atlas datasets with the Ensembl Gene ID and provided the Gene Name; Gene Synonym; Gene Description; Protein Class; Molecular Function and Disease Involvement features in the individual gene expression page. The web-hosting Docker engine was utilized in an Ubuntu Linux server. All information regarding representative transcript expression is accessed with no restriction and is available at https://rttpg.ibms.sinica.edu.tw.

## Supplementary Information

Supplementary Information.

## Data Availability

All representative transcript expression data in human tissues can be accessed with no restriction by following link at https://rttpg.ibms.sinica.edu.tw.
