# Supplementary material for: Dominant transcript expression profiles of human protein-coding genes interrogated with GTEx dataset
Source: Sci Rep. 2022 Apr 28;12:6969. doi: 10.1038/s41598-022-10619-9 (PMC9050722; doi:10.1038/s41598-022-10619-9)
Supplement: Supplementary file 1 — Supplementary Information. [file 41598_2022_10619_MOESM1_ESM.docx]

**Dominant transcript expression profiles of human protein-coding genes interrogated with GTEx dataset**

Kuo-Feng Tung^1^, Chao-Yu Pan^1, 2^, and Wen-chang Lin^1, 2, *^

^1^Institute of Biomedical Sciences, Academia Sinica, Taipei, Taiwan, R.O.C.

^2^Institute of Biomedical Informatics, National Yang-Ming University, Taipei, Taiwan, R.O.C.

** Correspondence should be addressed to:* Wen-chang Lin. Institute of Biomedical Sciences, Academia Sinica, Taipei 115, Taiwan, R.O.C.
(email: wenlin@ibms.sinica.edu.tw)

Supplementary Table 1. Numbers and percentages of ranked transcripts with the Z-score of >3 in Rank1 to Rank10 classes.

|  | **Numbers of tissue representative transcripts  (Z-score > 3)** | **Total numbers of transcripts** | **Percentages of tissue representative transcripts** |
| --- | --- | --- | --- |
| **Rank1** | 15197 | 19591 | 77.6% |
| **Rank2** | 13350 | 16838 | 79.3% |
| **Rank3** | 11880 | 14934 | 79.6% |
| **Rank4** | 10473 | 13183 | 79.4% |
| **Rank5** | 9237 | 11579 | 79.8% |
| **Rank6** | 8068 | 10036 | 80.4% |
| **Rank7** | 6639 | 8714 | 79.5% |
| **Rank8** | 6043 | 7487 | 80.7% |
| **Rank9** | 5256 | 6432 | 81.7% |
| **Rank10** | 4422 | 5451 | 81.1% |

Supplementary Table 2. Average expression TPM values of tissue representative transcripts in Rank1 to Rank10 classes.

| **Tissues**  **Ranks** | **Zero Tissue**  **(Z-score<3)** | **One Tissue**  **(Z-score>3)** | **Two Tissues**  **(Z-score>3)** | **Three Tissues**  **(Z-score>3)** | **Four Tissues**  **(Z-score>3)** |
| --- | --- | --- | --- | --- | --- |
| **Rank1** | 91.05 | 23.23 | 14 | 9.95 | 8.69 |
| **Rank2** | 8.89 | 6.14 | 4.43 | 3.35 | 0.9 |
| **Rank3** | 4.77 | 2.85 | 2.31 | 1.57 | 1.27 |
| **Rank4** | 2.75 | 1.77 | 1.46 | 0.84 | 0.73 |
| **Rank5** | 2.02 | 1.16 | 1.04 | 0.61 | 0 |
| **Rank6** | 1.65 | 0.8 | 0.82 | 0.94 | 0.42 |
| **Rank7** | 1.32 | 0.62 | 0.63 | 0.68 | 0.18 |
| **Rank8** | 1.03 | 0.55 | 0.47 | 0.41 | 0.03 |
| **Rank9** | 0.91 | 0.45 | 0.37 | 0.3 | 0.08 |
| **Rank10** | 0.8 | 0.37 | 0.35 | 0.29 | 0.17 |

**
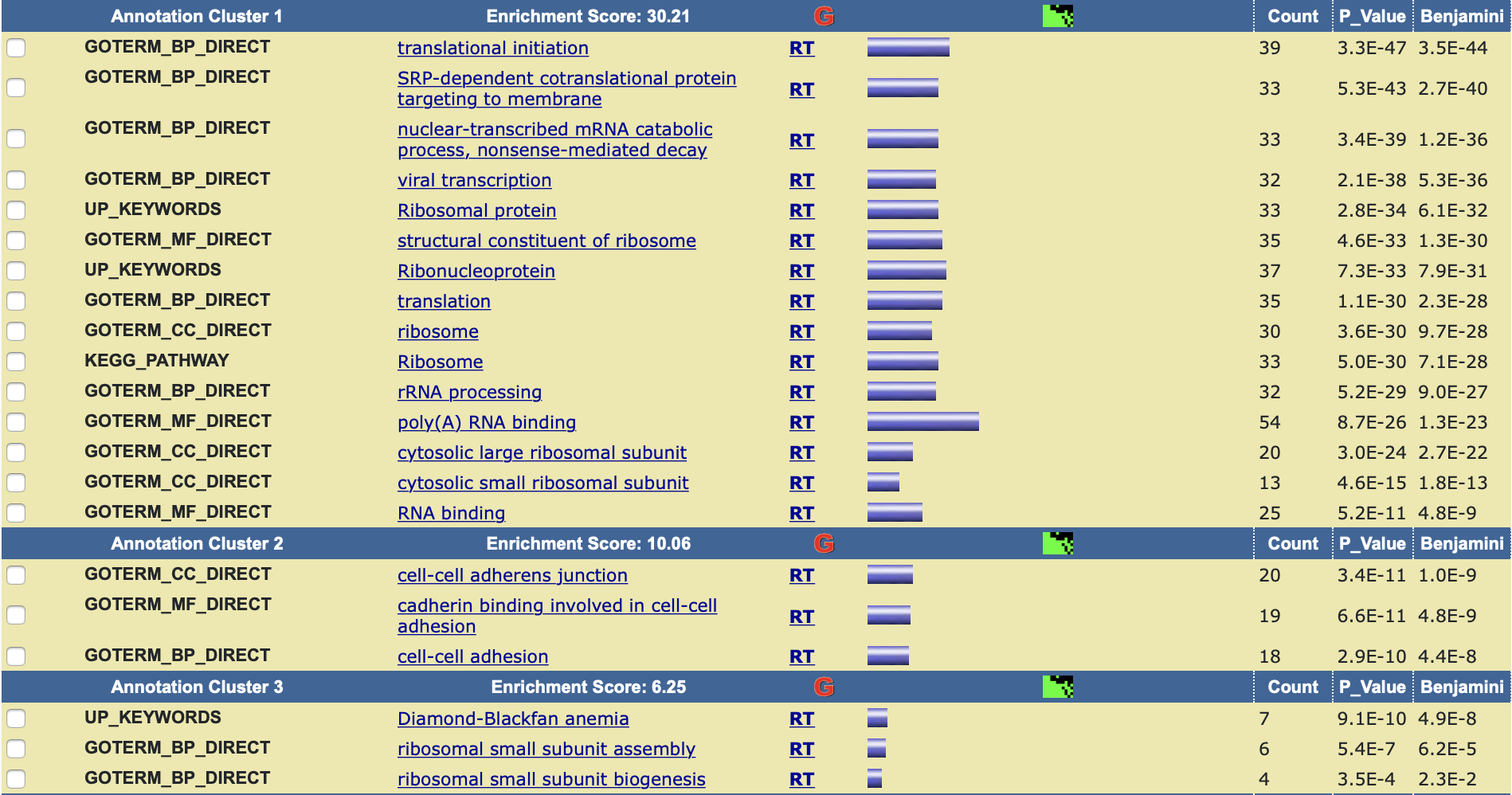
**

**Supplementary Figure 1.** DAVID pathway function enrichment analysis for ubiquitously expressed protein-coding genes. These include genes with a Z-score values of <3 for all transcripts. Among them, selected protein-coding genes with high expression levels (the average TPM value >100) were uploaded to the DAVID web analysis pipeline. The top enrichment cluster is the translation process-related pathway.

**
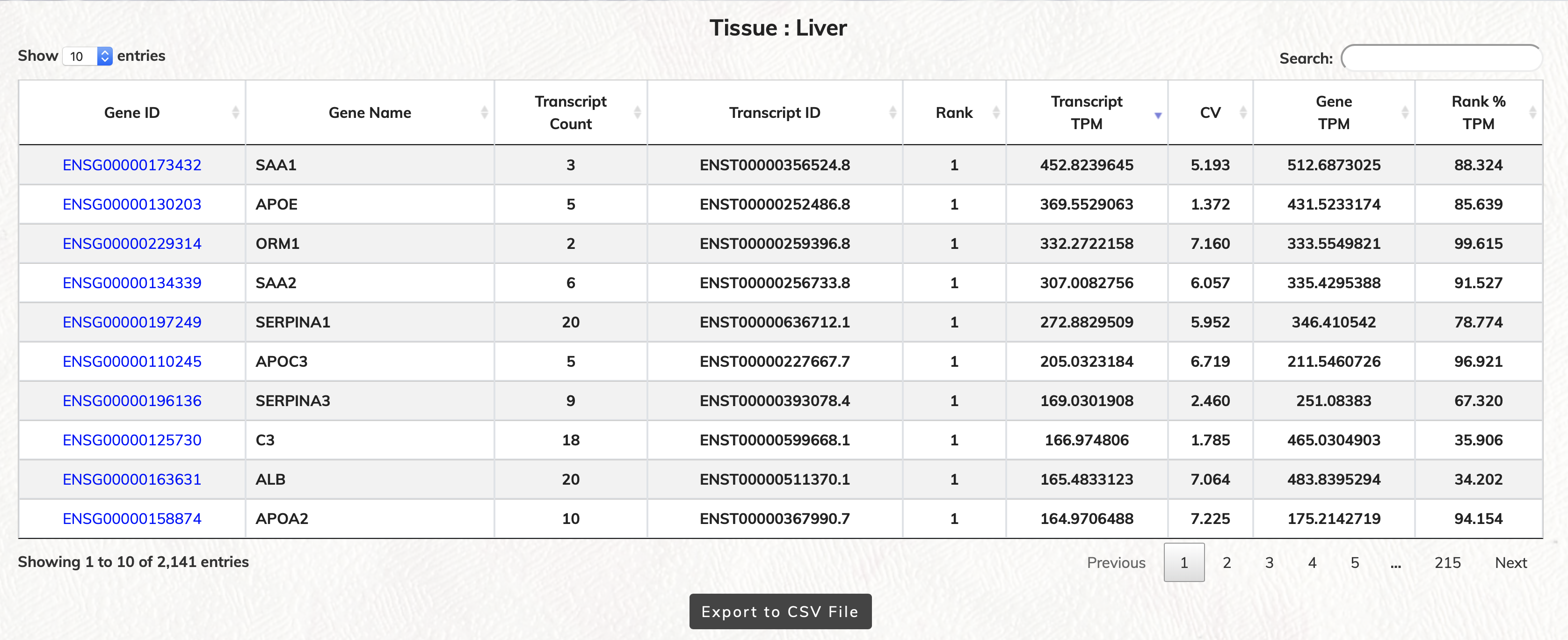
**

**Supplementary Figure 2A**

**
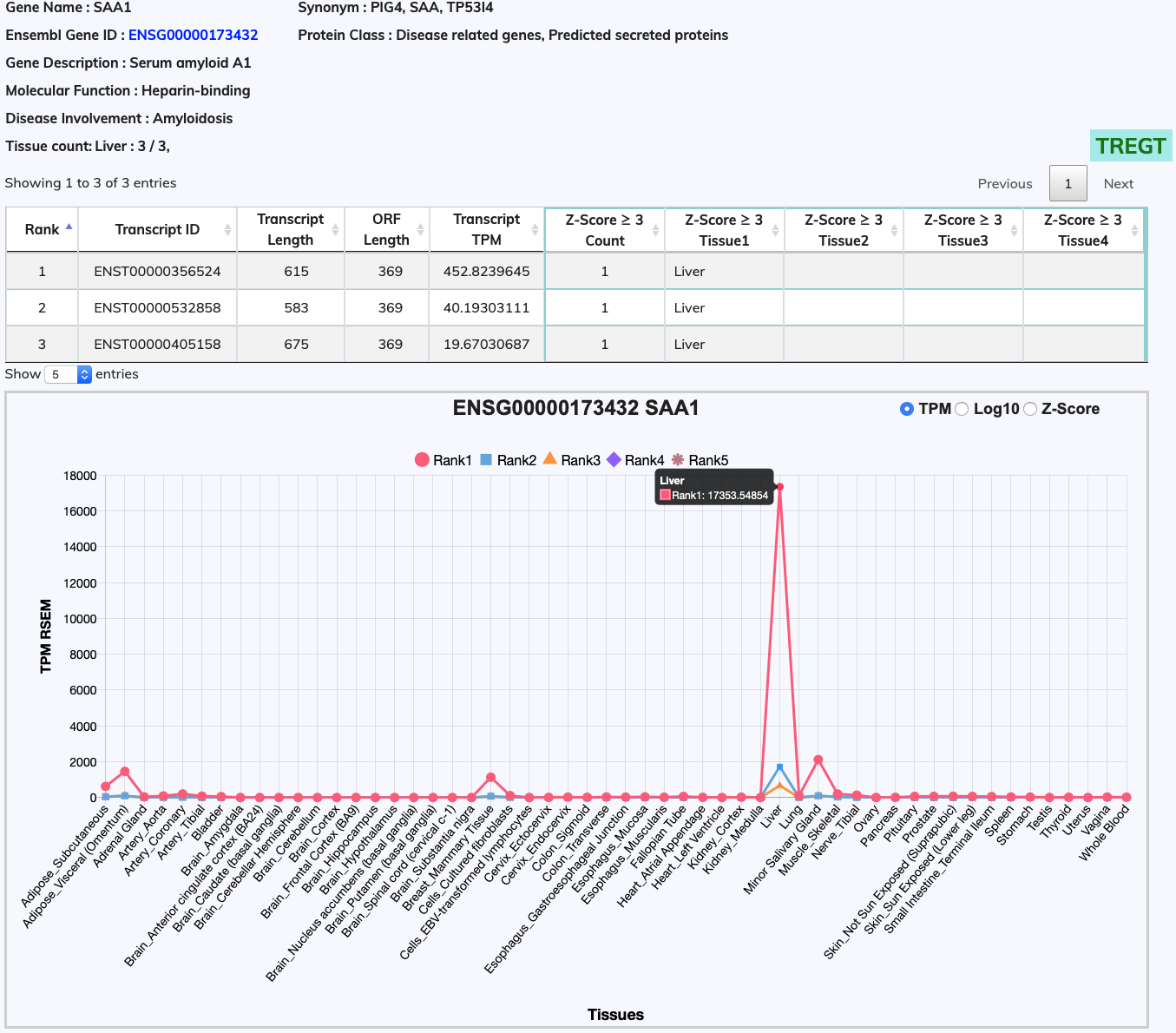
Supplementary Figure 2B**

**Supplementary Figure 2.** Web user interfaces for the RTTPG database. (A) List of tissue representative transcripts in the liver tissue with a Z-score of >3. Features listed in the table were gene ID; gene name; transcript count; transcript ID; rank of that particular transcript; transcript TPM value; CV; gene TPM value; and rank transcript expression percentage in gene. (B) The serum amyloid A1 protein gene (SAA1) has three transcripts, which are all highly expressed in the liver tissue. In this web page, additional descriptions on the gene and function are shown on the top including gene name, synonym; gene ID; protein class; gene description; molecular function; disease involvement. The representative tissue types and amounts are summarized in tissue count. In the transcript list table, individual transcript information regarding its expression rank; transcript ID; transcript length; ORF length; average expression TPM value; and Z-score >3 representative tissue types are illustrated. When mouseover any given datapoint, a pop-up text will display the tissue name of this datapoint as well as the expression TPM value or Z-score of the Rank transcript. Users can analyze the expression information by using TPM or Z-score values, and a Log button are available to change the TPM expression scale to log scale for observing lowly expressed transcripts. The TREGT link button on the right corner will open to the web page for additional transcript expression information.
